# Cast from the Past? Microbial Diversity of a Neolithic Stone Circle

**DOI:** 10.3390/microorganisms12112338

**Published:** 2024-11-16

**Authors:** Mercedes Martín-Cereceda, Amaya de Cos-Gandoy, Richard A. J. Williams, David Elliott, Andrea Serrano-Bellón, Blanca Pérez-Uz, Abel Sanchez-Jimenez

**Affiliations:** 1 Department of Genetics, Physiology and Microbiology, Faculty of Biology, Complutense University of Madrid, 28040 Madrid, Spain; richwill@ucm.es (R.A.J.W.); andrserr@ucm.es (A.S.-B.); perezuz@ucm.es (B.P.-U.); 2 Department of Biodiversity, Ecology and Evolution, Faculty of Biology, Complutense University of Madrid, 28040 Madrid, Spain; amayadec@ucm.es (A.d.C.-G.); abelsanchez@bio.ucm.es (A.S.-J.); 3 Nature Based Solutions Research Centre, University of Derby, Derby DE22 1GB, UK; d.r.elliott@derby.ac.uk

**Keywords:** Arbor Low, neolithic monument, microbiome, cyanobacteriota, pseudomonadota, euryarcheota, chlorophyta, spatial connectivity, methanogens, nitrogen-oxidizing archaea

## Abstract

We studied the microbial diversity colonizing limestone rock pools at a Neolithic Monument (Arbor Low, Derbyshire, England). Five pools were analyzed: four located at the megaliths of the stone circle and one pool placed at the megalith at the Gib Hill burial mound 300 m distant. Samples were taken from rock pool walls and sediments, and investigated through molecular metabarcoding. The microbiome consisted of 23 phyla of bacteria (831 OTUs), 4 phyla of archaea (19 OTUs), and 27 phyla of microbial eukarya (596 OTUs). For bacteria, there were statistically significant differences in wall versus sediment populations, but not between pools. For archaea and eukarya, significant differences were found only between pools. The most abundant bacterial phylum in walls was Cyanobacteriota, and Pseudomonadota in sediments. For archaea and microbial eukarya, the dominant phyla were Euryarcheota and Chlorophyta, respectively, in both wall and sediments. The distant pool (P5) showed a markedly different community structure in phyla and species, habitat discrimination, and CHN content. Species sorting and dispersal limitation are discussed as mechanisms structuring the microbiome assemblages and their spatial connectivity. The Arbor Low microbiome is composed of terrestrial representatives common in extreme environments. The high presence of Cyanobacteriota and Chlorophyta in the Arbor Low stones is troubling, as these microorganisms can induce mechanical disruption by penetrating the limestone matrix through endolithic/chasmoendolithic growth. Future research should focus on the metabolic traits of strains to ascertain their implication in bioweathering and/or biomineralization.

## 1. Introduction

The role of microbial populations in nature is crucial for ensuring the productivity, trophic stability, and resilience of habitats to disturbances and climate change [1]. Global biodiversity and conservation schemes cannot be comprehensively understood without exploring the microbial landscape. Yet, the management of both geo- and bio-heritage tends to focus exclusively on the larger plants and animals because they are easily visible and have public appeal for funding.

In geological habitats, microorganisms occupy numerous niches. They can make virtually any surface their home and adapt to different niches through the versatility of their metabolisms and lifestyles (benthonic and/or biofilm) [2]. Although this premise could sound like a movie quote of the style “life always finds a way”, at times it can be a real problem because of biological weathering. Some lithobiotic microorganisms help to shape the landscape by weathering [3,4]. Stone monuments are subject to physical and chemical processes that lead to their deterioration, above all the effects of water, wind, and heat. Microorganisms participate in stone monument deterioration in many ways: creating aesthetic (discoloration) problems, using the stone as a substrate, triggering physical stress (by penetration of filamentous organisms within the stone), or providing compounds for secondary chemical reactions such as acids (both organic and inorganic) and osmolytes, the last of which produced in response to changes in water activity which act as protectants against freezing, excessive heat and drying, salts, acids, and other factors [5]. There is also growing evidence that microbial organisms can be equally used for the bioremediation of these bioweathering processes, especially for stone consolidation through the inoculation of carbonatogenic bacteria [6,7]. Stone monuments may additionally be exposed to a catalog of contaminants that can potentially augment weathering. These include deposits from burning fossil fuels, organic compounds used in agriculture, heavy metals from industry, and other pollution from the air and precipitation. These may cause significant damage to stone buildings and monuments [8,9,10].

The microbial diversity of stone monuments and its role in their biogeomorphology has received increasing scientific attention [11,12,13]. The characterization of stone-colonizing microbiomes is crucial to the identification of potentially beneficial or undesirable microorganisms and to plan future strategies that aim for conservation, or better, restoration [3,14]. The development of molecular tools provided a powerful way to analyze microbial diversity and its distribution in stone monuments at a grand scale. Sequence metabarcoding—particularly the sequencing of hypervariable regions of the small subunit of the rRNA gene—has grown into a routine and non-invasive tool to undertake large scale environmental inventories of microorganisms, particularly in cultural heritage monuments from miscellaneous places. The walls of King Tutankhamun’s tomb [15], the Memphis necropolis of Egypt [16], petroglyphs of the Negev Desert [17,18], ancient Roman towns [19], 9th century World Heritage sites [3], Mayan pyramids [20], medieval churches and cathedrals [2,21,22,23], neoclassical (18th–19th century) granite churches [24], the walls of a historic 19th century townhouse [25], buildings from the former Auschwitz II–Birkenau concentration camp [26], and contemporary outdoor sculptures [27], among others, have been analyzed in detail by next-generation sequencing (NGS).

Further non-high-throughput genotyping has been accomplished for different microorganisms from ancient monuments, for example: black yeast in the oldest Egyptian pyramid of Giza [28], cyanobacteria in Cambodian temples [29], actinobacteria/cyanobacteria and black fungus in Roman hypogeal houses built in 1st Century BC [30], and a variety of bacterial strains from ancient Greek sculptures [31]. However, to our knowledge, next-generation sequencing has not been used to concurrently analyze the prokaryotic and eukaryotic microbiome of prehistoric stone monuments, and the microbial biodiversity of these ancient sites is therefore still largely unknown.

Arbor Low is a Neolithic limestone monument (2500 BC to 1500 BC), with statutory protection since 1882, formed by a stone circle and barrow (Arbor Low henge) and a large burial mound (Gib Hill double barrow) situated in the Carboniferous limestone plateau of the Peak District National Park (Derbyshire, UK). The monument is formed by horizontally positioned limestone slabs [32]. In early 2007, English Heritage assessed the Arbor Low monument as at “medium” risk and in “declining” condition. As a result of conservation work, their condition was judged to be “improving” in the last Conservation Plan [33]. The recumbent limestone slabs have eroded along the millennia and created a series of irregularly deep rock pools containing sediment and rainwater.

Rain-fed (ombrotrophic) freshwater rock pools, usually made of granite, sandstone, and sometimes limestone, constitute temporary and fluctuating habitats dependent on the unpredictability of precipitation or flooding rates [34,35]. The global distribution, structural simplicity, stability in geological time scale, and open exposure to climate change, make rock pools a model habitat for biological colonization [35,36,37]. These small freshwater habitats can be used for testing a variety of ecological hypotheses, and although they remain little explored at the microbial level, the limited research carried out to date reveals the high heterogeneity of microbial communities in freshwater rock pools [34,38,39,40,41,42,43]. However, to our knowledge, microbial composition and their ecological drivers have not been studied in rock pools from prehistoric monuments.

The objective of this work is to reveal, for the first time, the microbial diversity (bacteria, archaea, and eukaryotes) in both the limestone rock pool walls and sediments of the Arbor Low prehistoric stone monument, and to put their spatial connectivity in context. Due to the regular presence of livestock, which may transport microorganisms, we hypothesize strong connectivity between the microbial communities found in pools located at the Arbor Low stone circle, with a high species exchange among these pools. We also predict that stone walls and sediments will have very different microbial communities with the presence of species adapted to live in biofilms occurring more in the walls than in the sediments.

Limestone is one of the rock types with higher bioreceptivity and is susceptible to biodeterioration [44]. This manuscript is part of a comprehensive and long-term research project on this historic monument whose ultimate aim is to identify the species potentially involved in both the biodeterioration (bioweathering) of the prehistoric stones and in their conservation (biomineralisation), as well as to ascertain the microorganisms that regulate these microbial populations through predation (mainly bacterivorous protozoa).

Balanced microbial communities are essential for the global health of any ecosystem, but microorganisms can also be responsible for disrupting and worsening ecosystem wellness and stability. Therefore, the primary biodiversity analysis presented here provides a baseline that feeds subsequent applied research. Informed bioconservation decisions require knowing first which microorganisms are in an ecosystem and how they are distributed, in order to understand the role that they play in ecosystem stability and environmental health.

The scientific and community benefits of this microbial biodiversity inventory will include the following: (a) providing an empirical database for the surveillance and monitoring of species in a prehistoric geoheritage site at a widely unknown and invisible scale; (b) adding to the understanding of the natural landscape that supports the very base of the ecosystem; (c) highlighting the positive value of microorganisms and helping to define local policies for historic heritage conservation and management; (d) identifying microorganisms which can contribute to sustainable farming, land management, and nature recovery; (e) identifying microbial biodeteriogens and developing restoration strategies.

## 2. Materials and Methods

### 2.1. Study Area

**Figure 1:**
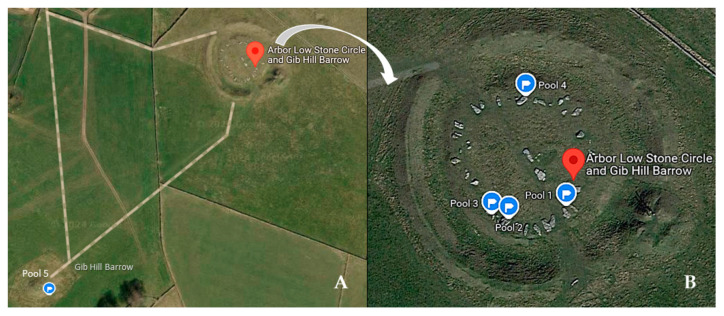
Arbor Low monument in the Derbyshire Peak District National Park (England, UK). (**A**) Stone circle and Gib Hill Barrow; P5: Pool 5 located at Gib Hill; (**B**) Location of pools P1 to P4 in the stone circle image adapted from Google Maps. Imagery © 2024 Google, Maxar Technologies, Map Data ©2024.

Arbor Low is a Neolithic monument located in the Peak District National Park (Derbyshire, UK, 53°10′08.7″ N 1°45′41.9″ W). The monument (Figure 1) consists of a stone circle of about 50 limestone blocks/slabs, which have now mostly toppled and have a horizontal orientation and a burial mound (Gib Hill barrow). The length of the slabs varies between 1.6 m and 2.9 m [33]. Gib Hill is thought to be a Neolithic oval barrow with an Early Bronze Age round barrow superimposed at one end. It is located about 300 m south-west of the Arbor Low stone henge. No trees or bushes are present at the monument area. Livestock (cows and sheep) are often found on and around the stone circle as this is placed in farmland. A fence separating the stone circle from the burial mound prevents the stock from reaching the Gib Hill mound. The recumbent blocks are very irregular in form and are deteriorated. They were quarried from limestone pavement and often have solution holes and cracks which go fully through the stone [33]. The surfaces have patchy patterns of clearly visible lichens, fungi, and mosses. It has been suggested that the stones could have been brought from a distance, as their color did not match the white limestone in the ditch. However, no experiments have been carried out to ascertain the origin of the stones [33].

### 2.2. Characterization of the Rock Pools and Collection of Samples

**Figure 2:**
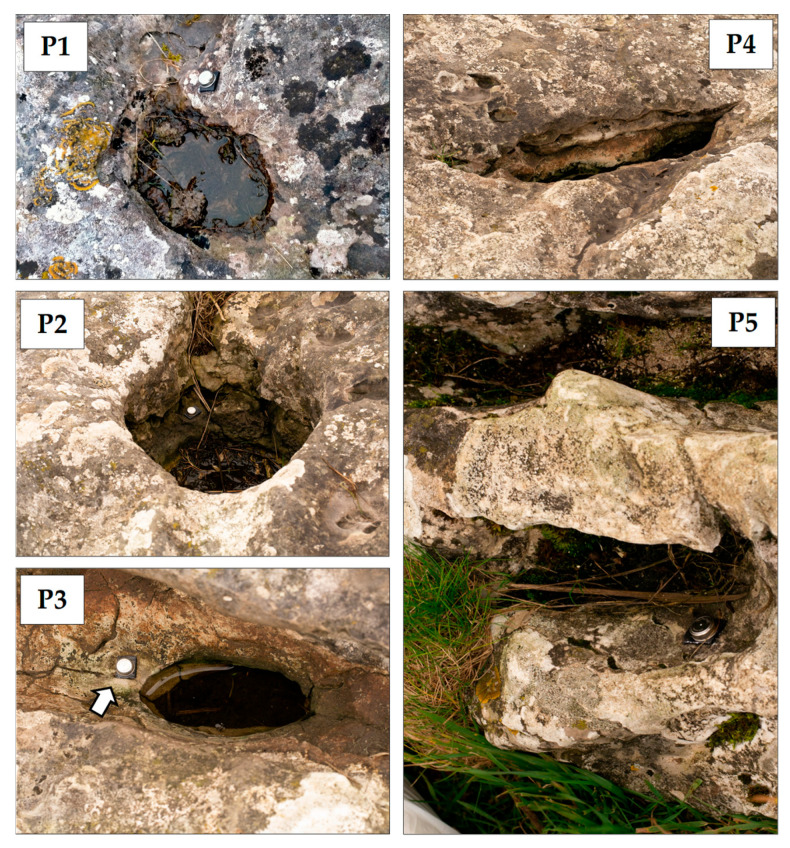
Rock pools sampled in Arbor Low. Arrow shows the e-button data logger in P3 (see Materials and Methods for more details on the e-buttons). P1: Pool 1; P2: Pool 2; P3: Pool 3; P4: Pool 4; P5: Pool 5.

**Figure 3:**
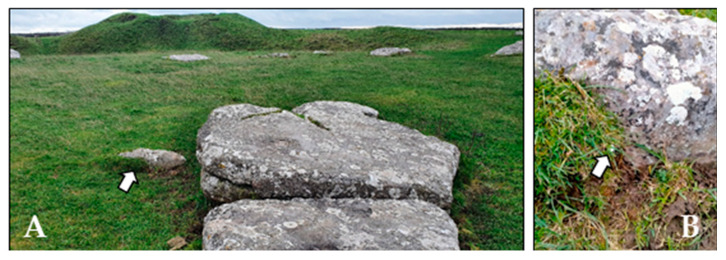
Location of soil sample. (**A**) Arbor Low central cove of stones. Arrow points to the stone where the soil sample (P0) was taken. (**B**) Detail of the area where P0 was collected. Arrow points to the e-button logger at P0 sampling site.

Five pools were selected for this study (hereafter P1 to P5; Figure 1 and Figure 2). P1 to P4 in the stone circle and P5 in the burial mound of the monument (Gib Hill). To provide a reference of the microbial soil community in the area, we also took a sample from the top 2 cm of soil (P0), at the center of the stone circle, where a smaller “cove” of stones is located (Figure 3). The area is well drained, with silty soil of the Malham 2 association [33] covered with short, wet, animal-grazed grass. Fine plant roots were removed from the sample using laboratory sterile tweezers before extracting DNA.

For each of the five pools, two types of samples were collected in November 2022: (i) from the rock wall surface using sterile 5 BD SWUBE dual cotton swabs with wooden stick and screw cap (BD, Franklin Lakes, NJ, USA), covering the entire diameter of the wall 5 cm above the water level; and (ii) from the sediment of each pool (approximately 5 g), using a sterile laboratory spatula after homogenizing the sediments. These were included in sterile 5 mL falcon. All samples were stored at 4 °C and frozen at −80 °C for later DNA analysis.

The coordinates of the sampling sites are as follows: P0: 53°10′08.0″ N 1°45′41.5″ W; 53.168889, −1.761538; P1: 53°10′07.7″ N 1°45′41.2″ W; 53.168803, −1.761444; P2: 53°10′07.6″ N 1°45′42.1″ W; 53.168767, −1.761700; P3: 53°10′07.6″ N 1°45′42.4″ W; 53.168780, −1.761779; P4: 53°10′08.8″ N 1°45′41.9″ W; 53.169100, −1.761626; P5: 53°10′00.6″ N 1°45′53.1″ W; 53.166823, −1.764752. Altitude: 370 m above sea level (a.s.l.) for P0 and P1 to P4 and 420 m a.s.l. for P5. Total area occupied by the pools sampled in the stone circle: 376.52 m^2^ (4052.79 ft^2^); total distance covered by the pools: 95.02 m (311.75 ft).

### 2.3. Extraction of DNA, Sequencing, and Bioinformatics

Total genomic DNA was extracted using the PowerSoil Pro^®^ DNA Isolation Kit (Qiagen, Hilden, Germany) according to the manufacturer’s instructions for the pool sediments. The protocol was modified slightly for the swab sample extraction. Briefly, the heads of swabs were cut off, placed in a PowerBead Pro tube (step 1) and solution CD1 was added. After homogenization in a vortex adapter (step 2) and centrifuging the tubes (step 3), the swab heads were removed from the tubes and stored at −80 °C. Steps 2 and 3 of the protocol were then repeated to maximize the amount of DNA extracted from the swabs.

The DNA extracted from both swab and sediment samples was sent to Macrogen, Inc. (Seoul, Republic of Korea) for amplification and sequencing. DNA amplification was performed using the following primers that target the small subunit of the rRNA gene: for eukaryotes: 18S V4F (5′-TCGTCGGCAGCGTCAGATGTGTATAAGAGACAGCCAGCASCYGCGGTAATTC-3′) and V4R (5′-GTCTCGTGGGCTCGGAGATGTGTATAAGAGACAGACTTTCGTTCTTGATYRATG-3′); for bacteria: 16S V3-V4F (5′-CCTACGGGNGGCWGCAG-3′) and V3-V4R (5′-GACTACHVGGGTATCTAATCC-3′); for archaea: 16S Arc 787F (5′-ATTAGATACCCSBGTAGTCC-3′) and 1059R (5′-GCCATGCACCWCCTCT-3′). Sequencing analysis was performed using MiSeq 300 bp PE 1 Lane (Illumina Inc. (S. Diego, CA, USA), assembly with FLASH (1.2.11), pre-processing, and clustering with CD-HIT-OTU. The sequences were taxonomically classified using the curated NCBI database. Sequences with 97% similarity (cutoff 97%) were clustered to the same operational taxonomic unit (OTU). The OTU picking method was “de novo”.

### 2.4. Environmental and Chemical Analysis

The temperature (T, °C) and relative humidity (RH) of each pool (P1–P5) and of the soil sample (P0) were monitored for 24 h at all the rock pools using i-button^®^ sensors (DS1923 Hygrochron, Maxim Integrated Products, San José, CA, USA) placed at the walls on the pool immediately above the water level. These sensors record temperatures between −20 °C and +85 °C and relative humidities from 0 to 100% (accuracy of ±0.5 for temperature and 5% relative humidity). Measurements were recorded at 5 min intervals.

The carbon, hydrogen, and nitrogen (CHN) content of the sediments of each pool (P1–P5) and of the soil sample (P0) were determined with elemental microanalysis by combustion using a micro-analyzer LECO CHNS-932 (LECO Corporation. St. Joseph, MI, USA). Prior to the analysis, sediment was homogenized by pulverization until a final granulometry <125 µm was reached. The analysis was performed three times per sample.

### 2.5. Statistical Analysis

Venn diagrams (R package: VennDiagram; version: 1.7.3) [45] were used to relate both the common and the unique OTUs of the pools. The number of OTUs detected were plotted in rarefaction curves which were normalized by both the total number of reads and total number of OTUs (R package: iNEXT; version: 3.0.0) [46]. These analyses were performed for the sediment and the wall samples. Alpha diversity richness, Shannon–Weaver, and inverse Simpson diversity indexes were calculated based on raw reads (R package: vegan; version: 2.6-4). Hierarchical cluster analysis was performed using the hclust function by Ward’s minimum variance method [47]. Permutational Multivariate Analysis of Variance (PERMANOVA) tests were used to assess the differences in the composition of OTUs and phyla in the pools. Non-metric multidimensional scaling (NMDS) was applied to visualize comparatively the OTUs abundances (R package: vegan; version: 2.6-4). Indicator species analysis was used to determine the OTUs that best indicate the difference in the pools (R package: indicspecies, version: 1.7.13) [48]. Redundancy analysis (RDA) was performed to relate the abundance of the OTUs selected as indicator species to a matrix of chemical (explanatory) variables. OTU data were first transformed using the Hellinger transformation method. Significance was obtained by permutation tests (number of permutations: 999) for the whole model, the RDA axes, and the explanatory variables. The significance of the RDA axes and explanatory variables were examined further only if the overall RDA model was found to be significant after permutation. Linear dependencies within explanatory variables were verified using variance inflation factor, and forward selection was used in case of collinearity (R package: vegan 2.6-4) [49]. All analyses were performed with the RStudio software (vs 4.0.3) [47,50].

## 3. Results

### 3.1. Microbiome Global Structure: OTUs and Phyla

The bacterial community was represented by 831 OTUs covering 370,059 reads. The number of reads per pool ranged between 55,383 reads (P4) and 78,291 reads (P5). Far fewer bacteria (17,859 reads) were retrieved in the soil sample (P0). Regarding the number of OTUs, P2 had the highest number (513 OTUs) while P1 had the lowest number (381 OTUs). The richness of OTUs present in the soil (167 OTUs) was also much lower than in any of the pools.

Archaea were represented by only 19 OTUs. However, they accounted for a very high number of reads (308,513 reads). The abundance was considerably different among pools; P2 and P3 had 94,893 reads and 97,927 reads, respectively, while P1 and P5 had the lowest abundances (2043 reads and 7184 reads, respectively). The soil sample (P0) showed an intermediate abundance of archaea (35,398 reads). Archaeal OTUs were the highest in P4 (17 OTUs) and the lowest in P5 (only three OTUs).

Within microbial eukarya, 596 OTUs were found, representing a total abundance of 968,576 reads. The pool with the least reads was P1 (62,469 reads) and P2 was the pool with the highest abundance (273,371 reads). The soil sample (P0) had 113,425 reads. As for the OTU number, the soil sample (P0) had the highest OTU richness (326 OTUs), just opposite to that observed in soil for bacteria. As in bacteria, P2 was the pool with the highest number of OTUs (253 OTUs) and P1 the one with the least (187 OTUs).

**Figure 4:**
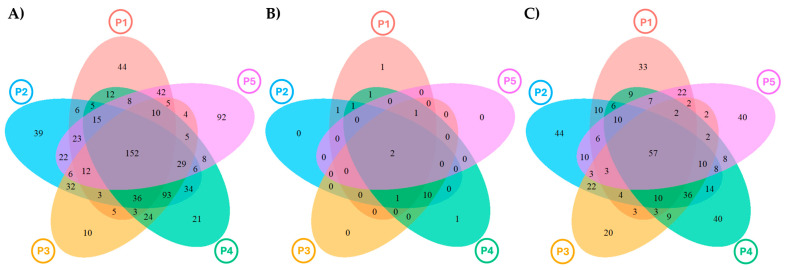
Venn diagrams showing the exclusive and shared OTUs retrieved in the five pools (P1 to P5). (**A**): Bacteria; (**B**): archaea; (**C**): eukarya.

A total of 18% (152 OTUs) of the bacterial OTUs were common to all the pools (Figure 4A), but few bacterial OTUs were exclusive to a single pool (between 1 and 5%), except in P5, where this percentage was almost double (Figure 4A). Between 40% and 60% of the bacterial OTUs found in each pool were exclusive to the wall, except in P5, where only 11% of the OTUs were wall-exclusive.

The number of archaeal OTUs common to all the pools was just 10.5% (2 OTUs) (Figure 4B). Three pools (P2, P3, and P4) did not have pool-exclusive OTUs. P1 and P4 had just one OTU exclusive to each pool (representing 5% of the total). Almost 100% of the archaeal OTUs found in each wall’s pool were also present in its sediment.

In eukaryotes, only 9.5% of the OTUs (57 OTUs) were common to all the pools (Figure 4C), and around 10% of OTUs were pool-exclusive, higher than reported for bacteria, except for Pool 3, where only 5% of OTUs were exclusive to this pool (Figure 4C). Similarly to that observed in bacteria, only 11% of the OTUs from P5 were wall-exclusive. In the other pools, the % of wall-exclusive OTUs varied appreciably by pool, from 21.8% in P3 to 54.5% in P1.

**Figure 5:**
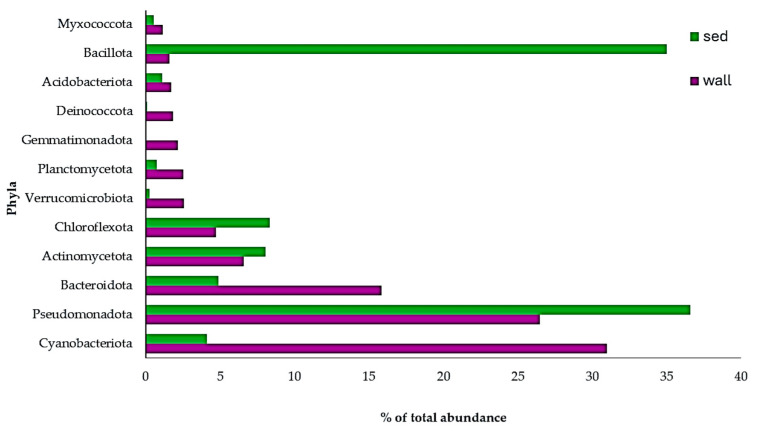
Relative abundance (%) of bacterial phyla in the wall and sediment (sed) of Arbor Low pools. Only phyla with a relative abundance higher than 1% are represented.

Bacterial OTUs were distributed across 23 phyla (Figure 5 and Table S1). Higher numbers of phyla were recovered from the wall than from the sediment. The lowest number of phyla was observed in the sediment of P1 (10 phyla) and in the soil (P0) sample (12 phyla). Each pool was more abundant in bacteria than the soil sample (P0). When discriminating wall versus sediment, there was approximately the same bacterial abundance in the walls than in the sediments (173,244 reads and 178,956 reads, respectively). In the walls overall, the most abundant phylum was Cyanobacteriota (53,672 reads; 31% of total), while in the sediments, Pseudomonadota (syn. Proteobacteria) and Bacillota (syn. Firmicutes) were the dominant phylum in abundance (65,468 reads; 36.6% of total and 62,647 reads; 35% of total, respectively) and Cyanobacteriota were only represented by 7339 reads (4.1% of total). The most abundant phylum in the walls of each pool was Cyanobacteriota too, except in P3, where Pseudomonadota was the most abundant. In the sediments, there was more heterogeneity by pool. In two pools (P1 and P4), the most abundant phylum was Bacillota, in two other pools (P2 and P3), Pseudomonadota was most abundant, and in P5, Cyanobacteriota was the most abundant phylum, occupying 97.7% of the total Cyanobacteriota in sediments. In the soil sample (P0), the most abundant phylum was Bacillota.

Archaeal OTUs were grouped into only four phyla (Table S2). In contrast to the findings observed in bacteria, the wall had less phyla than the sediment. The lowest number of phyla was in P1 and P5, in both walls and sediments (2 phyla). Archaeal wall abundance (28,871 reads) was much lower than that retrieved in the sediments (244,244 reads). The most abundant phylum in all pools combined was by far Euryarcheota (220,590 reads), with 90% of them present in the sediment samples. By pool, in P5, the most abundant phylum was Nitrosophareota (99.9% of total), while in the other pools, it was Euryarcheota. In the soil sample (P0), Nitrosophareota (35,197 reads; 99.4% of total) was the most abundant phylum.

**Figure 6:**
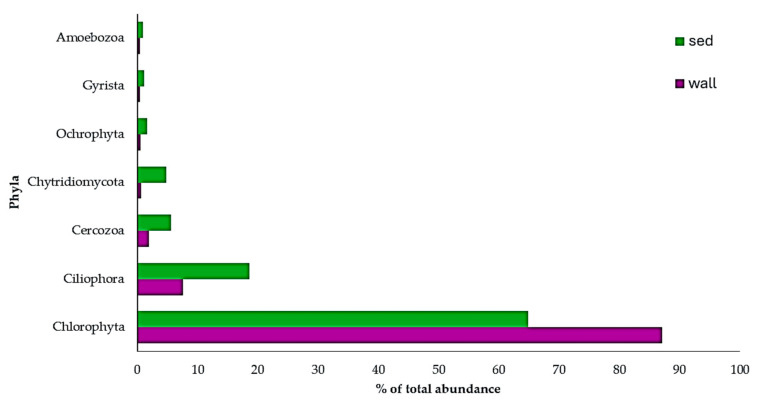
Relative abundance (%) of eukaryotic phyla in the wall and sediment (sed) of Arbor Low pools. Only phyla with a relative abundance higher than 1% are represented.

Eukaryotic OTUs were represented by 27 phyla (Figure 6 and Table S3). As we found for bacteria, a higher number of phyla were recovered from the wall than from the sediment, and the lowest number of phyla was found in the sediments of P1 (12 phyla). The number of phyla retrieved in the soil sample (P0), was, however, the highest (24 phyla) compared to any of the other pools. When discriminating wall versus sediment, there was lower eukaryotic abundance in the walls than in the sediments (366,059 reads and 489,092 reads, respectively). In the walls overall, the most abundant phylum was Chlorophyta (318,860 reads; 87.1% of total); the same was found in the sediment (317,566 reads; 65% of total). The most abundant phylum in the wall of each of the pools was also Chlorophyta, as well as in the sediments of P2, P3, and P5. In the sediments of P1 and P4, Amoebozoa were the most abundant. In the soil sample (P0), it was Apicomplexa (49,329 reads; 43.5% of total). The phylum Foraminifera only appeared in P0, albeit with a very low abundance (23 reads represented by just one OTU).

Tables S1–S3 detail the abundance (reads) and richness (OTU number) of each phylum of bacteria, archaea, and eukarya in both the walls and sediments of the five pools (P1–P5) and soil (P0) investigated.

### 3.2. Microbiome Alpha Diversity

**Figure 7:**
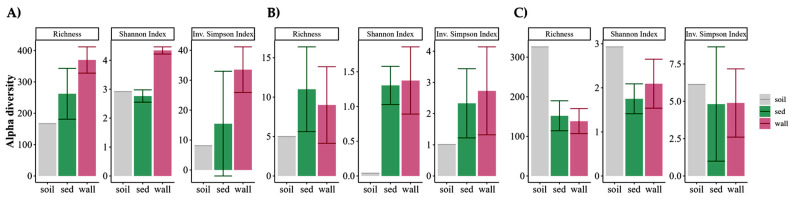
Box plots for OTU richness, Shannon, and inverse Simpson diversity indices in the overall pool wall and sediment (sed). (**A**): Bacteria; (**B**): archaea; (**C**): eukarya. Boxes represent the interquartile range and thick lines are the median. Whiskers indicate the highest and lowest values.

**Figure 8:**
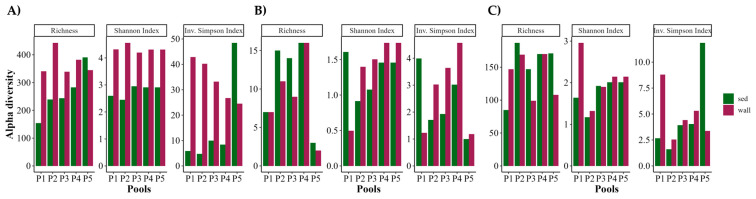
OTU richness, Shannon, and inverse Simpson diversity indices in wall and sediment (sed) per pool. (**A**): Bacteria; (**B**): archaea; (**C**): eukarya.

For prokaryotes (bacteria and archaea), the soil was the least diverse sample for both indexes (Shannon and inverse Simpson) (Figure 7A,B). It should be, however, noted that only one sample was analyzed for soil, while the represented diversity indexes for wall and sediment are the average of the five samples taken at each pool (Figure 7). The wall samples were much more diverse in bacteria than the sediment samples in all pools, except for P5, where the inverse Simpson index was much higher (Figure 8A). Archaea were also more diverse in the walls of all pools except for P1 (Figure 8B). In eukaryotes, contrary to the results observed in prokaryotes, the soil sample (P0) had higher diversity than the other pools, and OTU diversity was rather similar in wall and sediment samples (Figure 7C). By pool, there were only significant differences in eukaryotic diversity from wall to sediment in P1, which showed much higher diversity in the wall for both indices, and in P5, where sediment had a much higher inverse Simpson index than wall (Figure 8C).

**Figure 9:**
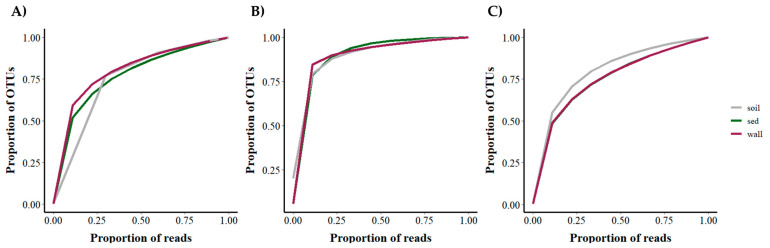
Normalized rarefaction curves of the number of OTUs in the soil, wall, and sediment (sed) samples. (**A**): Bacteria; (**B**): archaea; (**C**): eukarya.

The normalized rarefaction analysis showed very similar curves for wall and sediment. In bacteria and eukarya, with around 10% of the reads, about 50% of the OTUs had been retrieved (Figure 9A,C). In archaea, with around 10% of the reads, more than 75% of the OTUs had been recovered (Figure 9B). Therefore, there are very abundant OTUs within the three microbial groups, especially within the archaea.

### 3.3. Microbiome Beta Diversity

**Figure 10:**
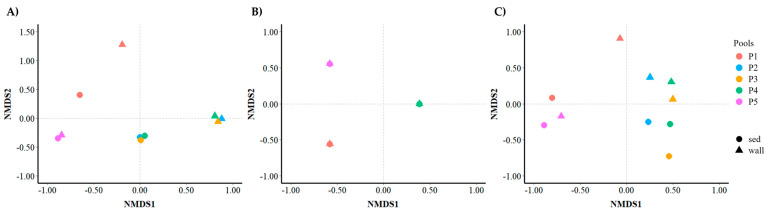
Bray–Curtis-based non-metric multidimensional scaling (NMDS) plot for the total OTUs of bacteria (**A**), archaea (**B**), and eukarya (**C**) in the wall and sediment (sed) samples.

In bacteria, non-metric multidimensional scaling (NMDS) OTU analysis showed that the axis NMDS1 ordinated by pool and axis NMDS2 by habitat type (wall versus sediment). For P5, wall and sediment are much closer to each other than was observed in the other pools (Figure 10A). PERMANOVA indicated that there were statistical differences in the composition of OTUs for the wall versus the sediment, but no differences were revealed between the pools (F-statistic: 3.1221; *p*-value: 0.001; F-statistic: 1.0436; *p*-value: 0.122, respectively). By contrast, for archaea, NMDS showed that there were no differences in the OTUs present in the wall versus sediment but revealed a clear separation of P1 and P5 from the rest of the pools (Figure 10B). PERMANOVA confirmed no differences between wall and sediment but differences among pools (F-statistic: 2.0376; *p*-value: 0.160; F-statistic: 9.9307; *p*-value: 0.002, respectively). In eukaryotes, NMDS did not show a clear ordination, but a gradient of pools could be observed along axis NMDS1, where P2, P3, and P4 were very similar and the sediment of P1 and both wall and sediment of P5 very different from the other pools (Figure 10C). PERMANOVA analysis did reveal differences between pools (F-statistic: 3.0992; *p*-value: 0.003) and showed differences very close to significance among wall versus sediment type of habitat (F-statistic: 2.6692; *p*-value: 0.053).

**Figure 11:**
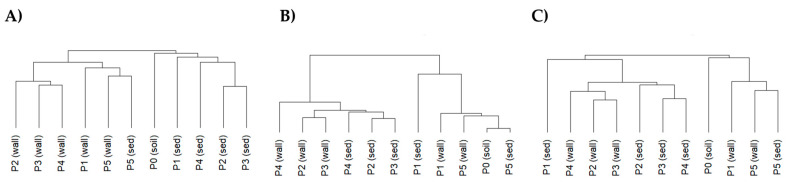
Hierarchical cluster of bacteria (**A**), archaea (**B**), and eukarya (**C**) per pool (P1 to P5) and type of sample: wall or sediment (sed). P0 represents the soil sample.

Cluster analysis corroborated NMDS results as the bacteria found in the sediment are clearly separated from those found in the walls, except for P5, where sediment and wall samples grouped together. Soil samples clustered plainly with the sediment pool samples (Figure 11A). For archaea, the clustering also agreed with NMDS results and showed two clear groups of pools: (P2, P3, P4) and (P1, P5), independently of wall or the sediment (Figure 11B). Cluster analysis for eukaryotes did not clearly separate wall samples from sediment samples either but showed a clear separation of P1 sediment and mainly P5 (both wall and sediment) from the rest of the pools (Figure 11C).

### 3.4. Microbiome Indicator Species

Indicator species analysis (ISA) was applied to reveal potential species associated either with wall/sediment and/or with pool. For bacteria, 59 OTUs were associated with wall and 11 OTUs were associated with sediment. Most of the wall indicator OTUs belonged to the phyla Bacterodiota, Pseudomonadota, and Cyanobacteriota, while the sediment indicator bacterial OTUs mainly clustered within Bacillota. No bacterial OTUs were recovered by the analysis as indicators of pool. In the case of archaea, no species were retrieved by ISA as pool indicator species neither for wall nor sediment. In eukaryotes, four OTUs were associated with wall and twelve OTUs with sediment. Most eukaryotic wall indicator OTUs belonged to the phylum Chlorophyta and the sediment indicator OTUs to the phyla Cercozoa and Apicomplexa. No eukaryotes OTUs were retrieved as indicators of pool. The taxonomic adscription of the indicator OTUs are compiled in Table 1 and Table 2.

### 3.5. Environmental Conditions of Arbor Low Rock Pools

The analysis of carbon (C), hydrogen (H), and nitrogen (N) (Table 3) showed that there are differences in the average content (n = 3) of sediment CHN among the pools, especially in the C content, which was significantly higher in the sediments of P1 than in the rest of pools. P5 values were most similar to the soil sample (P0).

Temperature and relative humidity were monitored for 24 h in the sampling day for all the pools and soil sample using e-buttons. The mean temperature was very similar in all the pools, slightly higher for P1, with an average of 11.22 °C. All the pools had a higher temperature than the soil sample (P0), which was 1 °C in average lower than that recorded in some of the pools (Table S4). The daily temperature had an oscillation between 4 °C and 6 °C for all the samples; the lowest temperature registered was 8.55 °C, and the highest was 14.54 °C, both recorded in P1 (Figure S1). The relative humidity (RH) of over 90% was very high, though this is normal in the rainy season in the area. RH was more variable among the pools than the temperature. A difference of up to 4% in average RH was registered, where P3 was the pool with the highest average RH (96.95%), and P4 the one with the lowest RH (92.88%). The soil sample (P0) showed slightly higher average values (97.11%) than P3 (Table S5). Daily RH values for the pools ranged more sharply than the temperature, with P3 and P1 recording the highest daily variation (about 20% of change) (Figure S2).

### 3.6. Associations Microbiome–Chemical Environment

**Figure 12:**
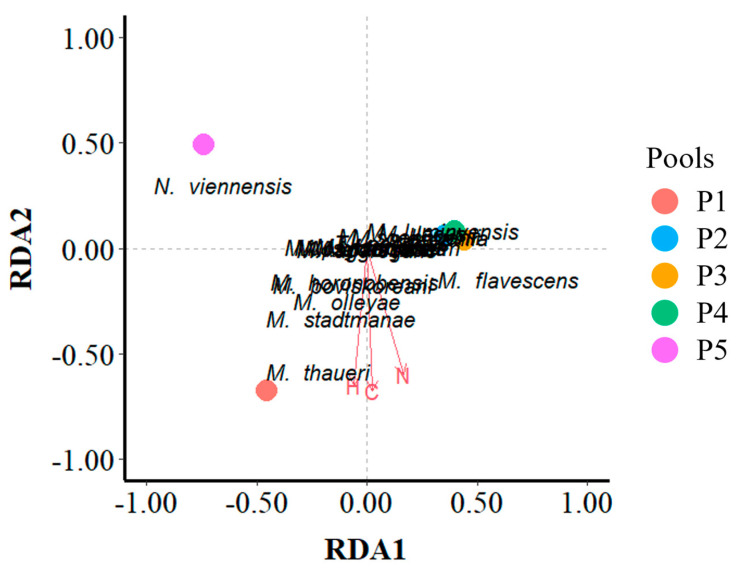
Redundancy analysis (RDA) for the OTUs of archaea (response variables) and the CHN sediment values (explanatory variables). All archaeal OTUs were included as ISA did not retrieve pool indicator species.

Redundancy analysis (RDA) was applied to evaluate the influence of the chemical variables of the sediment (CHN) on the variation in its OTU composition. Only for archaea the RDA model showed statistical significance. Axis 1 significantly (*p* = 0.025) discriminates among the pools and both constrained axes explain 94% of the variance observed (Figure 12). Therefore, almost all the differences observed in the Archaea community composition could be explained by the chemical profile, as CHN parameters showed high correlation among themselves. The methanogenic archaea *Methanobrevibacter thaurei* correlated with very high CHN values with its optimum in P1, and the ammonia-oxidizing archaea *Nitrososphaera viennensis* correlated with very low CHN values with its optimum in P5.

## 4. Discussion

### 4.1. Microbiome Global Distribution Patterns in the Rock Pools of a Neolithic Monument

Stone monuments located outdoors are openly exposed to the effects of physical, chemical, and biological factors. The inorganic surface of the stones facilitates the growth and development of a variety of microorganisms, creating a suitable habitat for them [2]. Our results show that the stones of the Arbor Low monument are colonized by the Domains bacteria, archaea, and microbial eukarya. However, several markers show that different dynamics exist in the colonization of walls and sediments, in the diversity indexes, and in the interconnection of the microbial populations in these three groups.

The bacteria colonizing this Neolithic site are significantly different in the walls and sediments of each pool, while no differences are observed among pools. By contrast, archaea and eukarya do not mirror this pattern. The community structure of these two last groups differs significantly by pool, but the habitat type (wall versus sediment) does not seem to affect it largely. Accordingly, the number of OTUs shared by all the pools is generally very low in archaea and eukarya communities (only around 10% of the total OTUs are common to all the pools), which translates into significant differences among the pools as revealed by NMDS, PERMANOVA, and cluster analysis. In bacteria, the higher percentage of OTUs that are common to all pools (18%) seems to be enough to maintain the pools under the same bacterial meta-community because no significant differences among the pools were retrieved by statistical analyses. As a whole, these results suggest that each individual pool is a more distinct habitat for archaea and microbial eukarya than it is for bacteria.

The analysis of soil from the Arbor Low monument site revealed that the soil, as expected, shared a more similar microbial community structure with the sediments than with the walls. Comparatively, the stone pools were a better niche for bacteria to thrive than for eukarya (OTU abundance ratio of 4.4 pool/soil in bacteria compared to 2.4 pool/soil, respectively). Moreover, the OTU richness ratio (pool/soil) was almost three times higher for bacteria than for eukaryotic microorganisms.

### 4.2. Indicator OTUs for Wall Versus Sediment

The Indicator Species Analysis (ISA) performed for bacteria showed that wall indicator OTUs largely belonged to Gram-negative bacteria of three main phyla: Bacteriodota, Pseudomonadota, and Cyanobacteriota. The first two phyla include representatives with a versatile range of metabolisms and habitats, while the third one (Cyanobacteriota) is wholly made of photoautotrophic bacteria. Several representatives of Bacteriodota belong to the Cytophaga-Flavobacteria group (*Cytophaga hutchinsonii*, *Spirosoma* spp., and *Flavobacterium* spp.), a group with gliding motility that enables the bacteria to move quickly over surfaces [51], or to other groups within the same phylum, i.e., *Chitinophagaceae*, which are also mucus-forming, and motile by gliding (*Mucibacter soli*; [52]). Many other OTUs cluster with species of bacteria which are typical of extreme environments, such as *Truepera radiovictrix*, an extremely ionizing radiation resistant strain [53], or with cryophilic species, including the following: *Abditibacterium utsteinense*, which was originally isolated from Antarctic oligotrophic soils and reported to be very resistant to toxins and antibiotics [54]; *Spirosoma arcticum*, collected from an arctic glacier [55]; *Hymenobacter tibetensis*, a high UV tolerant isolate from the Tibetan plateau [56]; *Luteolibacter arcticus* isolated from the Arctic tundra [57]; and *Actimicrobium antarcticum*, described in Antarctic coast waters [58]. The recent molecular detection of novel species in Arctic/Antarctic habitats has led to the hypothesis that an endemic polar microbiome exists [59]. Although this is very plausible, none of the aforementioned isolates can be described as endemic to the Polar Regions, as our study provides a new record for them in temperate zones.

Bacteria that develop on the walls/surfaces of outdoor monuments are described as mostly phototrophs and chemolithoautotrophs, which are characterized by relatively simple nutritional (inorganic minerals, atmospheric ammonia, etc.) and ecological needs (presence of light, CO_2_, and water) [2]. Our results show that Arbor Low rock walls are largely colonized by aerobic photosynthetic autotrophs as the most abundant and diverse phylum was Cyanobacteriota (31% of total abundance). This is in accordance with some previous works, among others: Li et al. (2016) [60], who found Cyanobacteria were the predominant phylum on the surface of stone sculptures accounting for approximately half of all bacteria communities; Macedo et al. (2009) [61], who indicated that limestone and marble display the greatest diversity of cyanobacteria and green algae within different lithotypes; Bolivar-Galiano et al. (2020) [62], who showed that Cyanobacteria were the second-most abundant group, after the green algae, on the stone surfaces from the fountains in the Alhambra, in Granada; and Nir et al. (2019) [17], who reported Cyanobacteria as the predominant phylum in petroglyphs from the Negev Desert. However, these results also differ, at least partially, to reports by other authors, as Coehlo et al. (2021) [21] and Skipper et al. (2022) [23] found that the dominant phylum on the limestone surfaces of cathedrals/churches in Portugal and England, respectively, was Actinobacteria, though Cyanobacteria also had a relevant presence.

Cyanobacteria are able to both photosynthesize and fix atmospheric nitrogen. Moreover, they are highly adapted to desiccation and to UV radiation [63]. Terrestrial cyanobacteria represent an important part of global bacterial diversity in different terrestrial habitats settling on rocks, caves, and modern and ancient stone buildings [64,65]. Some have been assigned a potential role in the bioweathering of stones through the production of biofilms, acids, discoloration, and endolithic growth [64] (and references therein). In Arbor Low, the wall indicator OTUs within the phylum Cyanobacteria represent a versatile array of species. *Sodaleptolyngbya stromatolitii* is a strain originally isolated from the surface of a stromatolite [66]; *Aliterella chasmolithica*, an isolate first found in crevices on granite rock boulders in the Atacama Desert [67]; *Macrochaete lichenoides*, an endosymbiont within the lichen *Placynthium* [68]; *Plectolyngbya hodgsonii*, found until now only in Antarctic lakes [69]; *Komarekiella chia*, a species with a habitat preference on calcareous rocks of a terrestrial cave [70]; and *Timaviella circinata*, a subaerophytic lithophyte found in cavernicolous habitats [71]. However, to our knowledge, none of these indicator OTUs, or in effect, any other cyanobacterial OTUs (53 OTUs in total) retrieved from Arbor Low rock walls or sediments, have been previously experimentally demonstrated to have specific bioweathering capabilities.

Only one of the wall indicator bacterial OTUs clustered with a species that is clearly associated with stone: the actinobacteria *Modestobacter lapidis*, a strain first isolated from a deteriorated sandstone building [72], although no causal association with bioweathering was described.

Our results show Arbor Low rock sediments are mostly colonized by Pseudomonadota (36.6%) and Bacillota (35%), and the sediment indicator OTUs are dominated by Gram-positive bacteria belonging to the phylum Bacillota, which produce endospores, are largely resistant to desiccation, and can survive extreme conditions [73], such as the desiccation of rock pools. Some of these indicators are aerobic or facultative aerobes (*Aneurinibacillus danicus*, *Brevibacillus borstelensis*, *B. fluminis*, and *Paenibacillus flagellatus*), but some others are strict anaerobes from the Class Clostridia, such as *Pseudoclostridium* (*Clostridium*) *thermosuccinogenes* [74], and some were first isolated from methanogenic reactors (*Lutispora thermophila*, [75] and *Anaerotaenia torta* [76]). These anaerobic OTUs were never recorded in Pool 5 (P5) of Arbor Low.

It is interesting to highlight two of the sediment indicator bacteria. The thermophilic bacteria *Brevibacillus borstelensis*, which together with *Pseudoxanthomonas mexicana* is the most abundant bacteria in Arbor Low. This strain can adapt to wide fluctuations in temperature [77] and is known to use polyethylene as its sole carbon and energy source [78]; and *Lysinibacillus boronitolerans*, recently reported as an excellent species for inducing calcium carbonate precipitation and used in self-healing concrete [79].

Several works have demonstrated the existence of microbial carbonatogenesis mechanisms like photosynthesis, urea hydrolysis, anaerobic sulfides oxidation, and production of extracellular polymeric substances. Urea hydrolysis is the most widespread method for calcium carbonate precipitation by bacteria [80]. To our knowledge, except for *L. boronitolerans*, none of the sediment indicator bacteria found in our study have previously been described as conductors of microbially induced calcium carbonate precipitation (MICP) or tested positively for bioweathering ability.

The archaea community did not present any wall versus sediment indicator OTUs. This group showed by far the lowest OTU richness and diversity indexes. This is to some extent surprising as the great development of metagenomic sequencing techniques has resulted in the discovery of a multitude of new archaeal lineages in recent years, which are increasingly being widely detected in many environments [81]. However, the universal 16S rRNA gene primers are thought to fail to detect a significant fraction of currently known archaeal diversity. Therefore, the combination of specific archaea-targeting primers that better capture the current diversity of this group is a priority to decipher the archaeome. Herein, we used the archaeal primer pair 787F–1059R that covers all archaea from the superphylum “Asgard”, which has been revealed as a ubiquitous group worldwide [82]; further studies using additional specific archaeal primers are in prospect, in order to confirm the range of archaea in the Arbor Low Neolithic monument.

Most of the eukaryotic wall indicators are represented by OTUs clustering with species of Chlorophyta, the majority split equitably between the classes Chlorophyceae and Trebouxiophyceae. This phylum is indeed by far the dominant eukaryote phylum in walls (87% of the total abundance) and also in the sediments (65% of the total abundance). These results agree with previous works that stated that among the algae, Chlorophyta are the most frequently found on historic monument stones [83,84]. Other examples are the marble and limestone Alhambra fountains [58], the sandstones of old temples [85,86], and a variety of stone monuments at the Mediterranean basin [61].

The two predominant eukaryotic OTUs in our study are associated with the Chlorophyta species *Desmodesmus armatus* and *Halochlorella rubescens*, the first of which, *D. armatus*, has been twice identified as an agent of soft tissue infection in humans [87].

The three indicator Chlorophyta OTUs in Arbor Low rock walls, *Pseudendoclonium incrustans*, *Planophila laetevirens*, and *Symbiochloris* sp., present flagellated zoospores which facilitate their spread in the environment and are able to form biofilms [88,89,90], explaining why they are very ubiquitous in the wall pools. *P. laetevirens* has an additional interest as it has been recently proposed as a sustainable source of silver nanoparticles that can be used as a potent therapeutic agent against cancer and infectious disease [91]. We have not found any study testing experimentally the bioweathering ability of these indicator OTUs, nor for any of the 82 OTUs of Chlorophyta retrieved in Arbor Low. Although indirectly, their contribution to biodegradation may however be important as promoters of growth in other organisms and also by their mechanical effect on the stone through their endolithic/chasmoendolithic colonization.

Although the most abundant phylum in sediments was also Chlorophyta, the indicator species of sediments were mostly composed of known bacterivorous or potentially bacterivorous eukaryotes: several Cercozoa, a naked amoeba, and a large soil ciliated protozoon, *Rigidohymena quadrinucleata*. Cercozoa are a large phylum of physiologically and morphologically diverse protists which are found abundantly in all type of habitats (terrestrial, marine, and freshwater habitats) [92] which usually have high growth rates and efficient encystment capacities that allow them to be well adapted to rock pool environments. *Echinamoeba exudans* [93] is a small bacterivorous naked monopodial amoeba (limax type) with a life cycle alternating proliferative stages and dormant cysts. This ecomorphological guild allows the amoeba to withstand the adverse conditions that occasionally prevail in the stones and therefore to be also well adapted to these environments. The ciliate *R. quadrinucleata* is a species typical of terrestrial and semiterrestrial habitats [94] and to our knowledge not previously recorded as a colonizer on ancient stone monuments. Ciliophora (ciliates), common in soil ecosystems, were the most abundant protozoa in Arbor Low, having a relative abundance of 7.5% in wall samples and 5.6% in sediment samples, within the range (1.1–11.6%) reported by Zhang et al. (2024) [95] in the walls of an ancient sandstone temple.

Fungi are frequently observed on the surfaces of historic stones. Their effects on the degradation of stone can be both due to synergistic mechanical and chemical processes. The most important are as follows: production of organic acids, penetration beneath the stone surface through the hyphae, shrinking and swelling of the hyphae and polysaccharides, and providing an entry for water that can freeze and thaw in the stones [96]. Many fungal lineages display versatile ecological strategies to adapt and survive in environments with extreme temperatures, periods of low nutrient availability, prolonged desiccation, and solar irradiation [97]. Therefore, it is perhaps surprising that they appeared in Arbor Low rock pools with such a low abundance. Eight phyla of fungi were retrieved in our study (Ascomycota, Blastocladiomycota, Basidiomycota, Chytridiomycota, Cryptomycota, Hyphochytridiomycota, Mucoromycota, and Zygomycota) but all of them had a relative abundance of less than 0.1% of the total, except for Chytridiomycota, which represented 4.9% of the microbial eukaryotes in the sediments. Our results are in accordance with those found in our previous research [40] on similar rock pools systems of different composition (granite), where only Chytridiomycota had a noticeable abundance among the fungi, which was higher in the granite pools (8.5% of the total abundance). Chytridiomycota have motile, waterborne zoospores that made them resilient through desiccation periods prone to happen in the rock pools. The fact that we did not recover more fungi in this study may be also related to the use of universal primers for the metagenomic analysis, which may have hampered the retrieval of more molecular diversity of Chytridiomycota, and fungi in general, in the pools. Further studies with fungi double-marker gene approaches and specific fungi primers [98] will be tackled in the future.

### 4.3. Indicator OTUs for CHN Profile

Arbor Low is not a pristine habitat. The presence of varying quantities of carbon (C) and nitrogen (N) in the pools can be explained by two main contributors. On the one hand, Arbor Low is a much-visited historic and spiritual site, which periodically hosts large gatherings. Traces of anthropogenic activities are often found in the pools (e.g., tokens left by the visitors, pebbles brought from other locations, and people walking all over the stones). Secondly, Arbor Low is visited daily by livestock (cattle and sheep) which have free access and roam through the stone circle, but cannot access the distant burial mound (Gib Hill). This partially explains why the pool located at the burial mound (P5) is so different from the rest of the pools. P5 did not receive the input of CN from animal sources and, therefore, had the lowest CN content. P5 was closest in composition to the soil sample (P0). Indeed, the early processes of primary soil formation and the development of herbaceous vegetation are visible in the vicinity of this pool (Figure 2), potentially leading to the accumulation of soil microbiota. By contrast, P1, which is located at the entrance of the stone circle for livestock, had the highest CN content. Moreover, epilithic communities (lichens, possibly mosses) are clearly visible around P1. The presence of these organisms may have contributed to raising this pool carbon content, through the decomposition of lichen thalli or moss turfs. The presence of livestock may have a particularly strong effect on structuring the archaeal community, mainly the methanogenic archaea found in Arbor Low, as all ruminants harbor methanogens in their guts. It has been reported that methanogens related to species of the genus *Methanobrevibacter* are the most highly represented archaea in the gut of herbivores [99]. In our study of Arbor Low, RDA analysis revealed that the methanogen *Methanobrevibacter thaurei*, first isolated from cow feces [100], is an indicator of the high CN content of P1 sediments. By contrast, P5 was shown by RDA as the niche of optimum CN values for the ammonium and urea-oxidizing archaea *Nitrososphaera viennensis*, which performs the first step into nitrification by forming nitrite [101]. Thus, P5 contains fully aerobic sediments in contrast to the rest of pools. One of the by-products of these ammonium oxidation bacteria (AOA) is nitrous oxide (N_2_O), a potent greenhouse gas [102]. Therefore, the study of these AOA is of prime interest to understand the ways for N_2_O mitigation.

In summary, these results suggest that sediments of pools in the stone circle (P1 to P4) contain micro-niches for methanogens, as they are dominated by anaerobic archaea of the phylum Euryarcheota, class Metanonomicrobia. P5 sediments are, however, more oxygenated as the dominant phylum is Nitrosophareota.

### 4.4. Drivers of Community Assemblages

Freshwater rock/stone pools are ideal habitats to understand the relative importance of species sorting (habitat type and environmental filtering) and dispersal limitation (distance between pools) because they are detached niche units [41].

In our study, the type of habitat seems to have more effect as a driver of the bacterial community than dispersal limitation, because there were significant differences among sediments and walls but not between the pools. This corroborates the existence of local bacterial communities (a meta-community) connected by potentially interacting species through dispersal, and plausibly structured by the livestock roaming through the pools and facilitating the exchange of bacteria from one pool to another. It should also be kept in mind that these pools are solely rain-fed, and therefore rainwater may be a source of microbiota; moreover, microbial airborne passive dispersers can arrive randomly at the pools through the wind, besides arriving through animal (livestock) vectors. For archaea, habitat type discrimination (wall/sediment) cannot be driving the OTU distribution as we have proposed for bacteria, because there were no statistically significant differences between sediment and wall populations. However, significant differences between the pools did exist. The same rationale based on the livestock, rain, and wind assisting the dispersal of populations applies to any other microbial population (archaea and eukarya); therefore, dispersal limitation should not be the reason explaining the differences, at least between the pools located in the stone circle (P1 to P4). The existence of another dimension of species sorting (environmental filtering) attributed to the CHN content differences between the pools, as shown by RDA, could explain the different distribution of archaea populations in the pools. In the case of eukarya, multivariate CHN RDA models were not significant. Therefore, for eukaryotic microorganisms, a combination of complex biotic (competition or cooperation among populations) and non-biotic factors not measured in this study could explain the different species distribution. The extent to which species sorting dimensions are drivers of the eukaryotic community assemblages are still unexplored and their study deserves consideration.

Dispersal limitation can only be admitted as the driver of community distribution in the case of P5, as this pool is isolated from the others and livestock is excluded from this site. P5 is markedly different from the other pools in crucial microbiome indicators: habitat discrimination, diversity, lowest CHN content, and global structure of the community. In this pool, no statistical differences were found between wall and sediment for any of the three microbial groups; P5 microbiome sediments were more similar to that of P5 walls, P1 walls, and even to the soil sample (P0), than to any of the other pool sediments. In summary, the results observed for P5 could be attributed to the location of this pool at the burial mound of the monument, far apart from the other four pools that are located within the main stone circle, where random dispersal can to some level act to homogenize the dynamics of the microbial communities in the pools.

The investigation of Neolithic rocks and their biological community offers a solid foundation to understand the relationship between biogeological heritage, and it creates an inclusive approach to the conservation and study of these prehistoric sites. To our knowledge, the results shown here represent the first report on both prokaryotic and eukaryotic microorganisms and their patterns of distribution and diversity in prehistoric rock/stone pool habitats. High-throughput DNA sequencing has permitted a non-invasive and comprehensive characterization of the microbial communities in the ombrotrophic rock pools of the Neolithic monument of Arbor Low (Derbyshire, England). Furthermore, it is beginning to unveil the interactions between microorganisms and their abiotic and biotic environment.

Altogether, our results seem to indicate that the microbiome of Arbor Low is composed of terrestrial representatives commonly bound to extreme environments and not of specific species accountable for stone monument bioweathering or stone bio-consolidation. However, the relevant presence of Cyanobacteriota and Chlorophyta in Arbor Low stones stand as a concern as these microorganisms can penetrate the stone matrix to become endolithic, with the corresponding risk of inducing degradation of the limestone [103]. Future research should focus on a polyphasic approach involving isolation, culture, and metabolomics of prokaryotic and eukaryotic Arbor Low strains in order to ascertain the active microbial populations and to explore their potential role in bioweathering or carbonatogenesis in the limestone rocks of this prehistoric monument.

## Figures and Tables

**Table 1:** Indicator species analysis (ISA) of bacteria OTUs. Indicator values range from 0 to 1. The relative abundance (%) of the OTUs in the walls (W) and sediments (S) of the pools is shown. Asterisks denote *p*-value (<0.05).

Phylum	Family	Species	Indicator Value	Abund (W)	Abund (S)
*Abditibacteriota*	*Abditibacteriaceae*	*Abditibacterium utsteinense*	0.989	0.98 *	0.02
*Acidobacteriota*	*Vicinamibacteraceae*	*Luteitalea pratensis*	0.991	0.98 *	0.02
*Actinomycetota*	*Geodermatophilaceae*	*Modestobacter lapidist*	0.983	0.97 *	0.03
	*Micromonosporaceae*	*Micromonospora tarapacensis*	0.953	0.09	0.89 *
*Bacillota*	*Bacillaceae*	*Lysinibacillus boronitolerans*	0.985	0.03	0.91 *
	*Clostridiaceae*	*Lutispora thermophila*	0.894	0	1 *
	*Lachnospiraceae*	*Anaerotaenia torta*	0.894	0	1 *
	*Oscillospiraceae*	*Pseudoclostridium thermosuccinogenes*	0.894	0	1 *
	*Paenibacillaceae*	*Aneurinibacillus danicus*	1	0	1 *
	*Paenibacillaceae*	*Brevibacillus borstelensis*	1	0	0.97 *
	*Paenibacillaceae*	*Brevibacillus fluminis*	0.894	0	1 *
	*Paenibacillaceae*	*Paenibacillus flagellates*	0.894	0	0.74 *
*Bacteroidota*	*Chitinophagaceae*	*Ferruginibacter paludism*	0.976	0.95 *	0.05
	*Chitinophagaceae*	*Mucibacter soli*	0.998	1 *	0
	*Cytophagaceae*	*Aquirufa antheringensis*	0.89	0.99 *	0.01
	*Cytophagaceae*	*Cytophaga hutchinsonii*	0.929	0.86 *	0.14
	*Cytophagaceae*	*Rhabdobacter roseus*	1	1 *	0
	*Cytophagaceae*	*Rudanella lutea*	0.886	0.78 *	0.22
	*Cytophagaceae*	*Rudanella paleaurantiibacter*	0.894	1 *	0
	*Cytophagaceae*	*Spirosoma agri*	1	1 *	0
	*Cytophagaceae*	*Spirosoma arcticum*	0.993	0.99 *	0.01
	*Cytophagaceae*	*Spirosoma humi*	0.894	1 *	0
	*Cytophagaceae*	*Spirosoma rigui*	0.954	0.91 *	0.09
	*Flavobacteriaceae*	*Flavobacterium aquariorum*	0.983	0.97 *	0.03
	*Flavobacteriaceae*	*Flavobacterium phycosphaerae*	0.989	0.98 *	0.02
	*Hymenobacteraceae*	*Hymenobacter tibetensis*	0.996	0.99 *	0.01
	*Hymenobacteraceae*	*Pontibacter humi*	0.991	0.98 *	0.02
	*Lewinellaceae*	*Flavilitoribacter nigricans*	0.941	0.89 *	0.11
	*Sphingobacteriaceae*	*Anseongella ginsenosidimutans*	0.904	0.82 *	0.18
	*Sphingobacteriaceae*	*Pedobacter aquicola*	0.948	0.9 *	0.1
	*Sphingobacteriaceae*	*Pedobacter planticolens*	0.999	1 *	0
	*Sphingobacteriaceae*	*Solitalea canadensis*	0.893	1 *	0
	*Spirosomaceae*	*Fibrella aquatilis*	0.983	0.97 *	0.03
	*Spirosomaceae*	*Fibrivirga algicola*	1	1 *	0
*Bdellovibrionota*	*Pseudobdellovibrionaceae*	*Bdellovibrio bacteriovorus*	1	1 *	0
*Cyanobacteriota*		*Sodaleptolyngbya stromatolitii*	0.979	0.96 *	0.04
	*Aliterellaceae*	*Aliterella chasmolithica*	0.928	0.86 *	0.14
	*Calotrichaceae*	*Macrochaete lichenoides*	0.936	0.88*	0.12
	*Leptolyngbyaceae*	*Phormidesmis communis*	0.986	0.97 *	0.03
	*Leptolyngbyaceae*	*Plectolyngbya hodgsonii*	0.894	1 *	0
	*Nostocaceae*	*Komarekiella chia*	0.971	0.94 *	0.06
	*Oculatellaceae*	*Oculatella lusitanica*	0.994	0.99 *	0.01
	*Oculatellaceae*	*Timaviella circinate*	0.946	0.9 *	0.1
*Deinococcota*	*Trueperaceae*	*Truepera radiovictrix*	0.983	0.97 *	0.03
*Myxococcota*	*Polyangiaceae*	*Chondromyces pediculatus*	0.993	0.99 *	0.01
*Planctomycetota*	*Gemmataceae*	*Tuwongella immobilis*	0.894	1 *	0
	*Gemmataceae*	*Urbifossiella limnaea*	0.866	0.75 *	0.25
	*Pirellulaceae*	*Aureliella helgolandensis*	0.993	0.99 *	0.01
*Pseudomonadota*	*Acetobacteraceae*	*Acidicaldus organivorans*	0.965	0.93 *	0.07
	*Acetobacteraceae*	*Roseococcus pinisoli*	0.979	0.96 *	0.04
	*Acetobacteraceae*	*Roseomonas chloroacetimidivorans*	0.894	0	1 *
	*Acetobacteraceae*	*Roseomonas ponticola*	0.973	0.95 *	0.05
	*Caulobacteraceae*	*Brevundimonas variabilis*	0.939	0.88 *	0.12
	*Devosiaceae*	*Devosia confluentis*	0.964	0.93 *	0.07
	*Oxalobacteraceae*	*Actimicrobium antarcticum*	0.964	0.93 *	0.07
	*Paracoccaceae*	*Albimonas pacifica*	0.894	1 *	0
	*Paracoccaceae*	*Szabonella alba*	0.963	0.93 *	0.07
	*Rhizobiaceae*	*Ensifer collicola*	0.93	0.12	0.78 *
	*Roseobacteraceae*	*Rubellimicrobium mesophilum*	0.951	0.9 *	0.1
	*Sphaerotilaceae*	*Aquincola rivuli*	0.913	0.83 *	0.17
	*Sphingomonadaceae*	*Sphingomonas arenae*	0.998	1 *	0
	*Sphingomonadaceae*	*Sphingomonas gotjawalisoli*	0.974	0.95 *	0.05
	*Sphingosinicellaceae*	*Polymorphobacter fuscus*	0.971	0.94 *	0.06
	*Xanthobacteraceae*	*Labrys soli*	0.925	0.86 *	0.14
	*Xanthomonadaceae*	*Lysobacter oligotrophicus*	0.995	0.99 *	0.01
*Verrucomicrobiota*	*Verrucomicrobiaceae*	*Luteolibacter arcticus*	0.986	0.97 *	0.03
	*Verrucomicrobiaceae*	*Prosthecobacter algae*	0.894	1 *	0
	*Verrucomicrobiaceae*	*Prosthecobacter fluviatilis*	0.894	1 *	0
	*Verrucomicrobiaceae*	*Prosthecobacter vanneervenii*	0.894	1 *	0
	*Verrucomicrobiaceae*	*Roseimicrobium gellanilyticum*	0.983	0.97 *	0.03

**Table 2:** Indicator species analysis (ISA) of eukarya OTUs. Indicator values range from 0 to 1. The relative abundance (%) of the OTUs in walls (W) and sediments (S) of the pools is shown. Asterisks denote significance (*p*-value < 0.05).

Phylum	Family	Species	Indicator Value	Abun (W)	Abun (S)
*Apicomplexa*		*Aranciocystis muskarensis*	0.996	0	1 *
	*Eimeriidae*	*uncultured Eimeriidae 1*	0.968	0.01	0.21 *
	*Eimeriidae*	*uncultured Eimeriidae 2*	0.905	0	0.26 *
	*Monocystidae*	*Monocystis agilis*	0.997	0	0.67 *
*Cercozoa*		*Cercozoa* sp.	0.984	0.01	0.29 *
		*uncultured Cercozoa 1*	0.973	0.01	0.24 *
		*uncultured Cercozoa 2*	1	0	0.93 *
		*uncultured Cercozoa 3*	0.986	0.02	0.58 *
		*uncultured Cercozoa 4*	0.894	0	0.11 *
	*Heteromitidae*	*Heteromita globosa*	0.894	0.01	0.82 *
	*Rhogostomidae*	*Rhogostoma epiphylla*	0.986	0.98 *	0.02
*Chlorophyta*	*Kornmanniaceae*	*Pseudendoclonium incrustans*	0.997	1 *	0
	*Planophilaceae*	*Planophila laetevirens*	0.999	0.98 *	0.02
	*Trebouxiaceae*	*Symbiochloris* sp.	0.991	0.97 *	0.03
*Ciliophora*	*Oxytrichidae*	*Rigidohymena quadrinucleata*	0.99	0.05	0.95 *
*Amoebozoa*	*Echinamoebidae*	*Echinamoeba exundans*	0.894	0	1 *

**Table 3:** Average content (in % of the total) of carbon (C), hydrogen (H) and nitrogen (N) in the sediments of each pool and the soil sample.

Pool	C (%)	H (%)	N (%)
P0 (soil)	9.33 ± 0.26	1.9 ± 0.03	1.00 ± 0.01
P1	41.49 ± 0.13	5.86 ± 0.02	3.52 ± 0.00
P2	24.71 ± 0.30	3.37 ± 0.05	2.38 ± 0.03
P3	19.90 ± 0.03	2.52 ± 0.02	1.94 ± 0.04
P4	27.54 ± 0.35	3.98 ± 0.04	3.07 ± 0.01
P5	12.23 ± 0.05	1.95 ± 0.02	1.32 ± 0.01

## Data Availability

Taxonomic affiliation and Gen Bank accession numbers are available through the research platform Open Science Framework (OSF https://osf.io/vdspf/?view_only=cebd77e8480e41b8b5cac7be09b8c136 (accessed on 27 September 2024)). Project: Microbiome Neolithic site Peak District NP). Other data are available from the authors upon request.

## Supplementary Materials

The following supporting information can be downloaded at: https://www.mdpi.com/article/10.3390/microorganisms12112338/s1, Table S1: Abundance (reads) and number of OTUs of bacterial phyla in the pools (wall and sediment) and in the soil sample (P0); Table S2: Abundance (reads) and number of OTUs of archaeal phyla in the pools (wall and sediment) and in the soil sample (P0); Table S3: Abundance (reads) and number of OTUs of eukaryotic phyla in the pools (wall and sediment) and in the soil sample (P0); Table S4: Temperature (°C) average values for the five Arbor Low rock pools and the soil sample investigated. SD: Standard Deviation; Table S5: Relative humidity (RH) average values for the five Arbor Low rock pools and the soil sample investigated. SD: Standard Deviation; Figure S1: Values (°C) of temperature recorded for a 24 h period at elapsed times (5 min) for each pool (P1–P5) and the soil sample (P0); Figure S2: Values (%) of relative humidity (RH) recorded for a 24 h period at elapsed times (5 min) for each pool (P1–P5) and the soil sample (P0).
