# Supplementary material for: Cast from the Past? Microbial Diversity of a Neolithic Stone Circle
Source: Microorganisms. 2024 Nov 16;12(11):2338. doi: 10.3390/microorganisms12112338 (PMC11596248; doi:10.3390/microorganisms12112338)
Supplement: Supplementary file 1 [file microorganisms-12-02338-s001.zip › Supplementary Information _Martin-Cereceda et al.pdf]

Table S1. Abundance (reads) and number of OTUs of Bacteria Phyla in the pools (wall and sediment) and in the soil sample (P0).

Sample	**Variable**	**ABDI**	**ACI**	**ACTI**	**ARMA**	**BACI**	**BACTER**	**BDELL**	**CAMP**	**CAND**	**CHLAM**	**CHLORO**
P1 (wall)	Abun	431	140	1664	57	683	7000	195	0	0	17	8
	OTUs	1	5	47	1	20	65	3	0	0	2	4
P2 (wall)	Abun	90	451	3223	5	1363	3583	291	2	13	12	4301
	OTUs	1	6	63	1	44	83	5	1	1	1	10
P3 (wall)	Abun	26	233	2195	3	76	7724	24	3	209	9	823
	OTUs	1	3	40	1	20	78	5	1	1	1	10
P4 (wall)	Abun	16	224	2391	2	623	2194	10	10	1	55	2559
	OTUs	1	6	58	1	32	68	3	1	1	1	13
P5 (wall)	Abun	605	1947	1920	339	44	6945	12	0	0	2	471
	OTUs	1	8	57	1	8	75	2	0	0	1	6
P0 (soil)	Abun	0	6	2491	0	11297	24	0	0	0	0	9
	OTUs	0	2	57	0	47	5	0	0	0	0	2
P1 (sed)	Abun	0	0	5444	0	21572	1860	0	0	0	0	12
	OTUs	0	0	25	0	31	7	0	0	0	0	3
P2 (sed)	Abun	0	13	1343	0	14681	509	8	0	0	2	1598
	OTUs	0	1	43	0	69	9	1	0	0	1	10
P3 (sed)	Abun	1	57	1356	0	6015	278	4	0	22	1	8170
	OTUs	1	3	39	0	56	19	1	0	1	1	12
P4 (sed)	Abun	1	30	2322	3	16578	3247	1	0	9	39	1687
	OTUs	1	2	43	1	73	25	1	0	1	1	11
P5 (sed)	Abun	25	1854	3942	14	3801	2827	3	0	0	4	3436
	OTUs	1	9	80	1	28	72	1	0	0	1	9

ABDI, Abditibacteriota; ACI, Acidobacteriota; ACTI, Actinomycetota; ARMA, Armatimonadota; BACI, Bacillota; BACTER, Bacteroidota; BDELL, Bdellovibrionota; CAMP, Campylobacterota; CAND, Candidatus Melainabacteria; CHLAM, Chlamydiota; CHLORO, Chloroflexota; CYA, Cyanobacteriota; DEIN, Deinococcota; FIB, Fibrobacterota; GEM, Gemmatimonadota; IGNA, Ignavibacteriota; MYX, Myxococcota; PLAN, Planctomycetota; PSEUD, Pseudomonadota; RHOD, Rhodothermota; THERMOD, Thermodesulfobacteriota; THERMOM, Thermomicrobiota; VERR, Verrucomicrobiota.

Table S1 (cont). Abundance (reads) and number of OTUs of Bacteria Phyla in the pools (wall and sediment) and in the soil sample (P0).

Sample	**Variable**	**CYA**	**DEIN**	**FIBR**	**GEM**	**IGNA**	**MYX**	**PLAN**	**PSEUD**	**RHOD**	**THERMOD**	**THERMOM**	**VERR**
P1 (wall)	Abun	8908	786	0	204	0	72	360	8668	46	5	24	1554
	OTUs	28	1	0	3	0	4	9	132	1	2	1	12
P2 (wall)	Abun	10226	216	0	948	0	27	1014	9361	0	6	130	440
	OTUs	32	2	0	3	0	5	20	143	0	5	1	16
P3 (wall)	Abun	8811	29	0	1625	0	139	1184	10116	1	1	40	1440
	OTUs	27	1	0	3	0	6	18	108	1	1	1	12
P4 (wall)	Abun	6552	24	0	960	5	38	707	4949	0	57	146	234
	OTUs	30	1	0	3	1	9	16	117	0	4	1	15
P5 (wall)	Abun	19175	2125	3	19	0	1695	1084	12770	34	236	39	787
	OTUs	35	3	1	3	0	11	6	114	1	3	1	8
P0 (soil)	Abun	2	0	0	0	0	11	4	2020	0	50	22	1923
	OTUs	2	0	0	0	0	3	2	42	0	2	1	2
P1 (sed)	Abun	20	0	0	3	0	0	10	9693	0	0	18	6
	OTUs	7	0	0	2	0	0	3	72	0	0	1	3
P2 (sed)	Abun	69	0	0	3	0	135	171	23916	0	4	19	27
	OTUs	5	0	0	2	0	2	10	77	0	3	1	5
P3 (sed)	Abun	40	0	0	14	1	9	319	19803	0	11	27	27
	OTUs	5	0	0	2	1	2	10	82	0	3	1	5
P4 (sed)	Abun	44	1	0	9	0	703	187	8659	7	14	68	17
	OTUs	13	1	0	2	0	1	11	85	1	3	1	6
P5 (sed)	Abun	7166	131	0	33	0	110	659	3397	2	3	258	374
	OTUs	35	3	0	3	0	8	8	117	1	2	1	11

ABDI, Abditibacteriota; ACI, Acidobacteriota; ACTI, Actinomycetota; ARMA, Armatimonadota; BACI, Bacillota; BACTER, Bacteroidota; BDELL, Bdellovibrionota; CAMP, Campylobacterota; CAND, Candidatus Melainabacteria; CHLAM, Chlamydiota; CHLORO, Chloroflexota; CYA, Cyanobacteriota; DEIN, Deinococcota; FIB, Fibrobacterota; GEM, Gemmatimonadota; IGNA, Ignavibacteriota; MYX, Myxococcota; PLAN, Planctomycetota; PSEUD, Pseudomonadota; RHOD, Rhodothermota; THERMOD, Thermodesulfobacteriota; THERMOM, Thermomicrobiota; VERR, Verrucomicrobiota.

Table S2 Abundance (reads) and number of OTUs of Archaea Phyla in the pools (wall and sediment) and in the soil sample (P0).

Sample	**Variable**	**THERMOP***	**EURY**	**NITRO**	**THERMO**
P1 (wall)	Abun	0	70	631	0
	OTUs	0	6	1	0
P2 (wall)	Abun	2531	7861	1331	150
	OTUs	1	8	1	1
P3 (wall)	Abun	398	1498	149	5
	OTUs	1	6	1	1
P4 (wall)	Abun	2455	11066	347	355
	OTUs	1	13	1	1
P5 (wall)	Abun	0	2	22	0
	OTUs	0	1	1	0
P0 (soil)	Abun	0	201	35197	0
	OTUs	0	4	1	0
P1 (sed)	Abun	0	1056	286	0
	OTUs	0	6	1	0
P2 (sed)	Abun	9096	70031	2842	1051
	OTUs	1	12	1	1
P3 (sed)	Abun	12009	82488	635	745
	OTUs	1	11	1	1
P4 (sed)	Abun	7926	46514	1105	1300
	OTUs	1	13	1	1
P5 (sed)	Abun	0	4	7156	0
	OTUs	0	2	1	0

THERMOP*, Candidatus Thermoplasmatota; EURY, Euryarchaeota; NITRO, Nitrososphaerota; THERMO, Thermoproteota.

Table S3. Abundance (reads) and number of OTUs of Eukarya Phyla in the pools (wall and sediment) and in the soil sample (P0).

Sample	**Variable**	**AMO**	**APHE**	**API**	**ASCO**	**BACI**	**BASI**	**BIGY**	**BLAS**	**CERC**	**CHLO**	**CHOA**
P1 (wall)	Abun	1394	0	7	80	4	59	4	0	4007	6479	20
	OTUs	8	0	1	11	2	9	1	0	48	27	2
P2 (wall)	Abun	8	3	87	36	0	18	8	36	574	118155	74
	OTUs	5	1	7	6	0	5	1	1	42	50	2
P3 (wall)	Abun	4	52	845	2	5	9	104	50	661	81730	76
	OTUs	3	1	5	2	1	4	1	1	17	36	2
P4 (wall)	Abun	19	172	251	28	4	126	2	33	1092	69448	131
	OTUs	5	1	11	8	2	9	1	2	44	38	2
P5 (wall)	Abun	14	0	3	37	7	86	0	0	639	43048	7
	OTUs	3	0	1	6	1	8	0	0	24	36	1
P0 (soil)	Abun	746	0	49329	160	0	240	31	16	26274	1113	42
	OTUs	22	0	27	6	0	7	2	1	130	18	1
P1 (sed)	Abun	27859	0	171	1019	0	3975	12	0	10068	253	0
	OTUs	2	0	10	5	0	2	1	0	37	10	0
P2 (sed)	Abun	46	0	2223	174	0	78	254	104	5893	132636	90
	OTUs	8	0	12	5	0	2	1	1	75	36	2
P3 (sed)	Abun	14648	19	3289	3	0	11	2737	128	2747	108646	236
	OTUs	8	1	15	1	0	1	1	1	58	28	1
P4 (sed)	Abun	48483	24	2114	12	0	1282	17	377	2738	48250	41
	OTUs	6	1	18	1	0	2	2	2	69	32	1
P5 (sed)	Abun	6	0	66	181	3	118	3	0	2278	27781	42
	OTUs	3	0	5	6	1	10	1	0	49	49	2

AMO, Amoebozoa; APHE, Aphelida; API, Apicomplexa; ASCO, Ascomycota; BACI, Bacillariophyta; BASI, Basidiomycota; BIGY, Bigyra; BLAS, Blastocladiomycota; CERC, Cercozoa; CHLO, Chlorophyta; CHOA, Choanozoa; CHYT, Chytridiomycota; CILI, Ciliophora; CRYP, Cryptomycota; DINO, Dinophyta; ENDO, Endomyxa; FORA, Foraminifera; GYRI, Gyrista; HYPH, Hyphochytridiomycota; META, Metamonada; MUCO, Mucoromycota; OBAZ, Obazoa; OCHR, Ochrophyta; OOMY, Oomycota; PERK, Perkinsozoa; SANC, Sanchytriomycota; ZYGO, Zygomycota.

Table S3 (cont.). Abundance (reads) and number of OTUs of Eukarya Phyla in the pools (wall and sediment) and in the soil sample (P0).

Sample	**Variable**	**CHYT**	**CILI**	**CRYP**	**DINO**	**ENDO**	**FORA**	**GYRI**	**HYPH**	**META**	**MUCO**
P1 (wall)	Abun	132	239	882	0	0	0	816	0	0	59
	OTUs	7	9	3	0	0	0	8	0	0	1
P2 (wall)	Abun	934	3240	16	32	242	0	243	33	0	17
	OTUs	7	14	5	3	2	0	5	1	0	1
P3 (wall)	Abun	1011	16069	19	1	216	0	1	12	10	0
	OTUs	7	4	2	1	2	0	1	1	1	0
P4 (wall)	Abun	211	7959	103	2	0	0	10	66	202	13
	OTUs	9	16	4	1	0	0	3	1	2	1
P5 (wall)	Abun	58	123	0	22	0	0	436	0	0	0
	OTUs	6	8	0	1	0	0	8	0	0	0
P0 (soil)	Abun	3105	1137	325	27675	575	23	134	0	9	955
	OTUs	23	24	15	1	6	1	4	0	1	11
P1 (sed)	Abun	126	3405	165	3	0	0	6	0	0	0
	OTUs	2	9	3	1	0	0	3	0	0	0
P2 (sed)	Abun	309	6169	21	1105	13	0	38	17	138	2
	OTUs	8	11	6	3	2	0	4	1	4	1
P3 (sed)	Abun	79	7681	24	67	5	0	29	229	306	0
	OTUs	3	10	2	2	2	0	1	1	3	0
P4 (sed)	Abun	72	7834	38	60	0	0	6	105	4218	0
	OTUs	7	13	4	1	0	0	2	1	2	0
P5 (sed)	Abun	315	2477	0	38	0	0	1816	0	0	0
	OTUs	5	21	0	1	0	0	8	0	0	0

AMO, Amoebozoa; APHE, Aphelida; API, Apicomplexa; ASCO, Ascomycota; BACI, Bacillariophyta; BASI, Basidiomycota; BIGY, Bigyra; BLAS, Blastocladiomycota; CERC, Cercozoa; CHLO, Chlorophyta; CHOA, Choanozoa; CHYT, Chytridiomycota; CILI, Ciliophora; CRYP, Cryptomycota; DINO, Dinophyta; ENDO, Endomyxa; FORA, Foraminifera; GYRI, Gyrista; HYPH, Hyphochytridiomycota; META, Metamonada; MUCO, Mucoromycota; OBAZ, Obazoa; OCHR, Ochrophyta; OOMY, Oomycota; PERK, Perkinsozoa; SANC, Sanchytriomycota; ZYGO, Zygomycota.

Table S3 (cont.). Abundance (reads) and number of OTUs of Eukarya Phyla in the pools (wall and sediment) and in the soil sample (P0).

Sample	**Variable**	**OBAZ**	**OCHR**	**OOMY**	**PERK**	**SANC**	**ZYGO**
P1 (wall)	Abun	1	1086	130	0	0	8
	OTUs	1	4	4	0	0	1
P2 (wall)	Abun	2	150	41	0	9	0
	OTUs	1	3	6	0	1	0
P3 (wall)	Abun	0	752	77	0	0	4
	OTUs	0	2	4	0	0	1
P4 (wall)	Abun	0	5	406	0	0	58
	OTUs	0	2	7	0	0	1
P5 (wall)	Abun	0	77	83	3	0	0
	OTUs	0	2	2	1	0	0
P0 (soil)	Abun	63	82	1208	7	12	164
	OTUs	2	4	13	1	1	5
P1 (sed)	Abun	0	0	0	0	0	0
	OTUs	0	0	0	0	0	0
P2 (sed)	Abun	88	0	15	0	0	0
	OTUs	2	0	3	0	0	0
P3 (sed)	Abun	12	30	63	0	0	2
	OTUs	1	2	4	0	0	1
P4 (sed)	Abun	0	0	79	0	0	14
	OTUs	0	0	4	0	0	2
P5 (sed)	Abun	2	357	374	5	0	0
	OTUs	1	1	7	1	0	0

AMO, Amoebozoa; APHE, Aphelida; API, Apicomplexa; ASCO, Ascomycota; BACI, Bacillariophyta; BASI, Basidiomycota; BIGY, Bigyra; BLAS, Blastocladiomycota; CERC, Cercozoa; CHLO, Chlorophyta; CHOA, Choanozoa; CHYT, Chytridiomycota; CILI, Ciliophora; CRYP, Cryptomycota; DINO, Dinophyta; ENDO, Endomyxa; FORA, Foraminifera; GYRI, Gyrista; HYPH, Hyphochytridiomycota; META, Metamonada; MUCO, Mucoromycota; OBAZ, Obazoa; OCHR, Ochrophyta; OOMY, Oomycota; PERK, Perkinsozoa; SANC, Sanchytriomycota; ZYGO, Zygomycota.

Table S4. Temperature (°C) average values for the five Arbor Low rock pools and the soil sample investigated. SD: Standard deviation.

Sample	Average Pool T ºC
P1	11,39
P2	11,17
P3	11,18
P4	11,16
P5	11,22
P1-P5 average	11,22
SD	0,08
Soil average	*10,98*

Table S5. Relative humidity (RH) average values for the five Arbor Low rock pools and the soil sample investigated. SD: Standard deviation.

Sample	Average pool RH (%)
P1	93,27
P2	94,61
P3	96,95
P4	92,88
P5	93,72
P1-P5 average	94,76
SD	1,45
Soil average	*97,11*

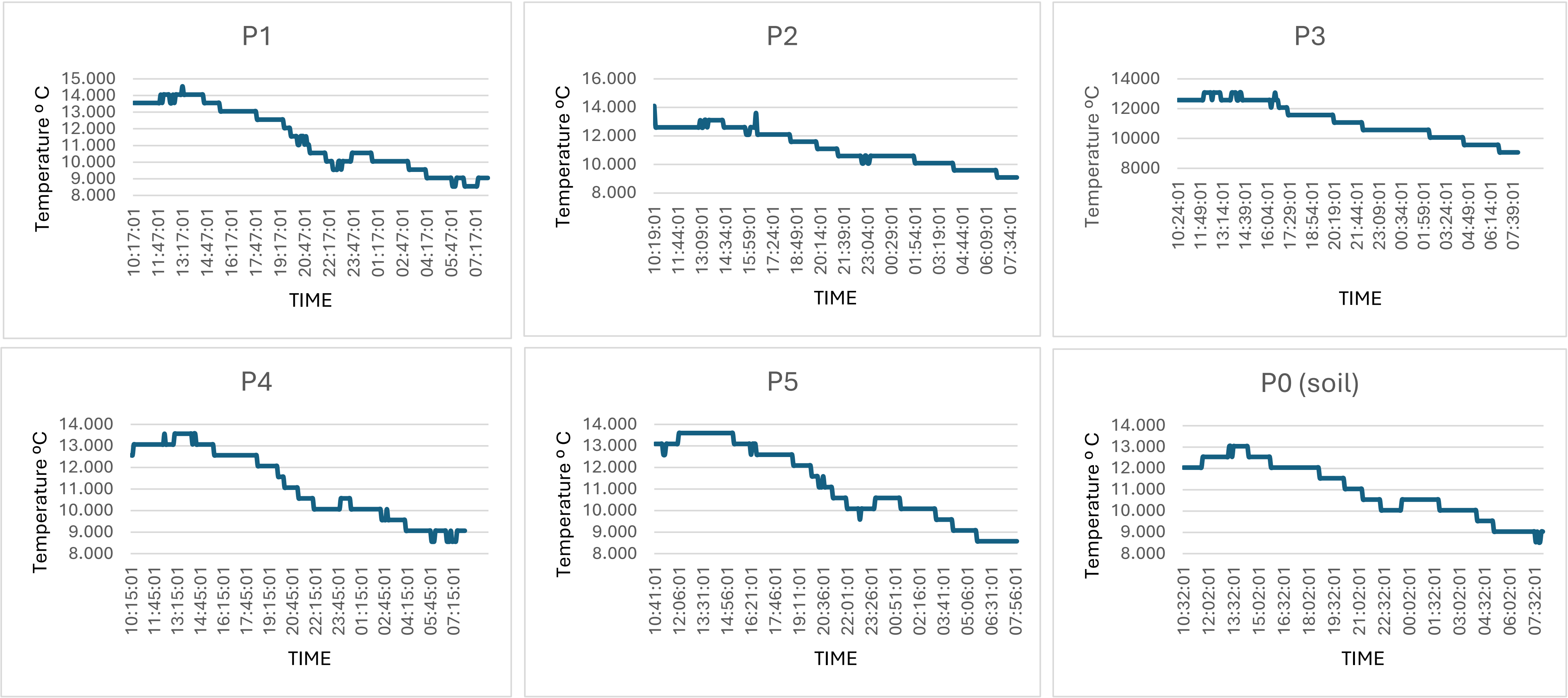

Figure S1. Values (^{o} C) of temperature recorded for a 24 h period at elapsed times (5 min) for each pool (P1-P5) and the soil sample (P0)

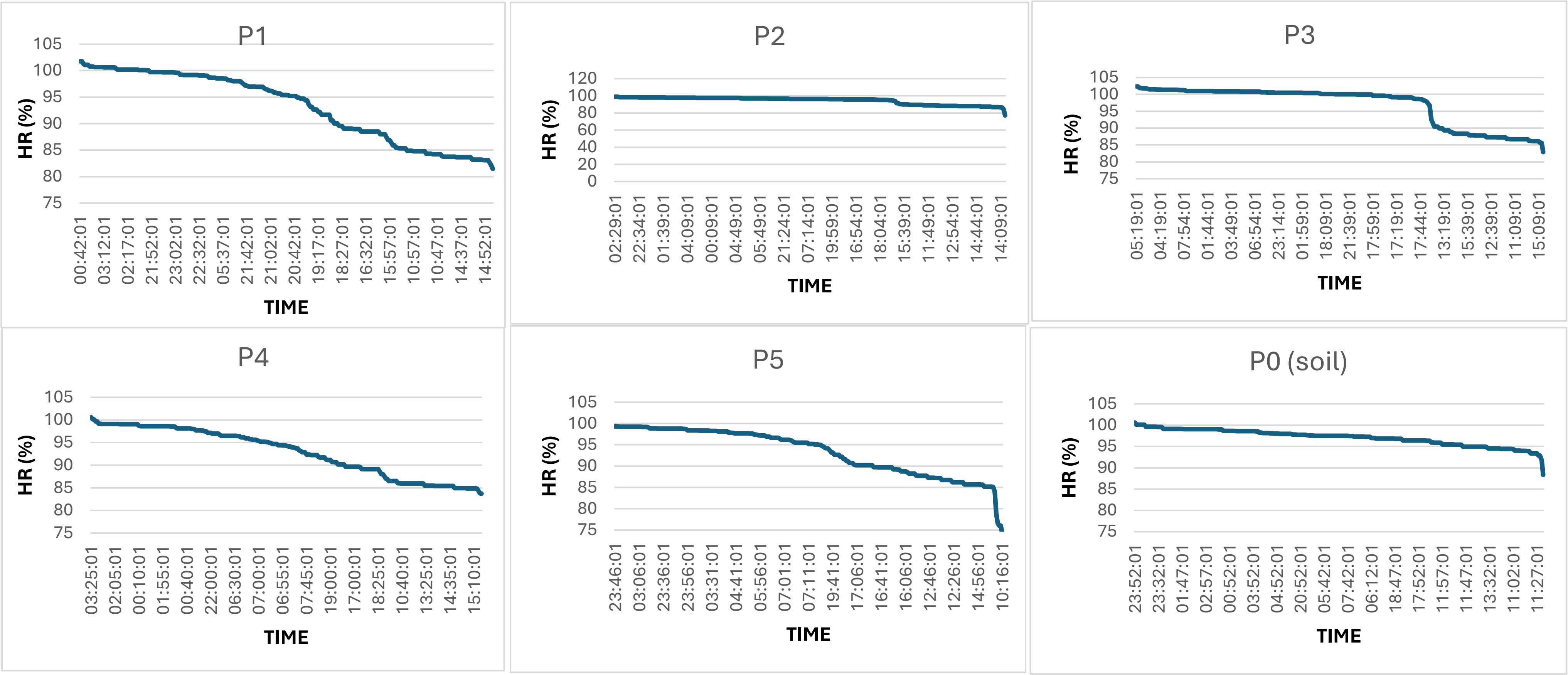

Figure S2. Values (%) of relative humidity (RH) recorded for a 24 h period at elapsed times (5 min) for each pool (P1-P5) and the soil sample (P0).
